# Fluid status assessment in heart failure patients: pilot validation of the Maastricht Decompensation Questionnaire

**DOI:** 10.1007/s12471-024-01921-4

**Published:** 2024-12-10

**Authors:** Arno J. Gingele, Fabienne Beckers, Josiane J. Boyne, Hans-Peter Brunner–La Rocca

**Affiliations:** Department of Cardiology, Maastricht University Medical Centre, Maastricht, The Netherlands

**Keywords:** Heart failure, eHealth, Prognosis, Risk assessment, Surveys and questionnaires, Validation study

## Abstract

**Background:**

eHealth products have the potential to enhance heart failure (HF) care by identifying at-risk patients. However, existing risk models perform modestly and require extensive data, limiting their practical application in clinical settings. This study aims to address this gap by validating a more suitable risk model for eHealth integration.

**Methods:**

We developed the Maastricht Decompensation Questionnaire (MDQ) based on expert opinion to assess HF patients’ fluid status using common signs and symptoms. Subsequently, the MDQ was administered to a cohort of HF outpatients at Maastricht University Medical Centre. Patients with ≥ 10 MDQ points were categorised as ‘decompensated’, patients with < 10 MDQ points as ‘not decompensated’. HF nurses, blinded to MDQ scores, served as the gold standard for fluid status assessment. Patients were classified as ‘correctly’ if MDQ and nurse assessments aligned; otherwise, they were classified as ‘incorrectly’.

**Results:**

A total of 103 elderly HF patients were included. The MDQ classified 50 patients as ‘decompensated’, with 17 of them being correctly classified (34%). Additionally, 53 patients were categorised as ‘not decompensated’, with 48 of them being correctly classified (90%). The calculated area under the curve was 0.69 (95% confidence interval: 0.57–0.81; *p* < 0.05). Cronbach’s alpha reliability coefficient for the MDQ was 0.85.

**Conclusions:**

The MDQ helps identify decompensated HF patients through clinical signs and symptoms. Further trials with larger samples are needed to confirm its validity, reliability and applicability. Tailoring the MDQ to individual patient profiles may improve its accuracy.

**Supplementary Information:**

The online version of this article (10.1007/s12471-024-01921-4) contains supplementary material, which is available to authorized users.

## What’s new?

- We developed a risk model based on expert opinion, which demonstrates diagnostic performance comparable to models that utilise more comprehensive datasets and advanced analytical methods.
- Readily available signs and symptoms of heart failure can aid in the identification of patients with decompensated heart failure.
- The Maastricht Decompensation Questionnaire is a useful tool that can be easily integrated into heart failure care pathways.

## Introduction

Heart failure (HF) is a serious condition, affecting approximately 1–3% of the general population [1]. HF management is costly, accounting for 1–2% of total healthcare expenditure in Western countries [2]. These expenses largely stem from frequent hospital admissions [3], with loop diuretics being the standard treatment for congested patients [4].

The primary factors contributing to decompensation are poor treatment adherence and delayed seeking of medical treatment by HF patients [5], and these issues result in up to 50% of readmissions being potentially preventable [6, 7]. However, the feasibility of closer monitoring of HF patients and optimising diuretic therapy is hindered by the growing shortage of healthcare professionals [8].

eHealth, the use of information and communication technologies in support of health and health-related fields [9], offers the potential to enhance HF care by empowering patients to manage their condition more autonomously. Although there is conflicting evidence regarding the impact of eHealth on clinical outcome [10–12], this discrepancy may be attributed to variations in available eHealth products and limitations in the studies conducted [13].

Nevertheless, identifying patients at risk of deterioration is crucial for adapting therapy and preventing further health deterioration [14]. Existing HF risk prediction models have exhibited limited performance [15], and eHealth devices used for remote monitoring often rely on risk models that were not tested or validated before being integrated into the devices [16, 17]. While artificial intelligence (AI) has shown promise in improving risk estimates, issues like overfitting and challenges in external validation persist [18]. Many of these models also necessitate various invasive and non-invasive data, which can impede their practical implementation in clinical settings [19].

**Fig. 1:**
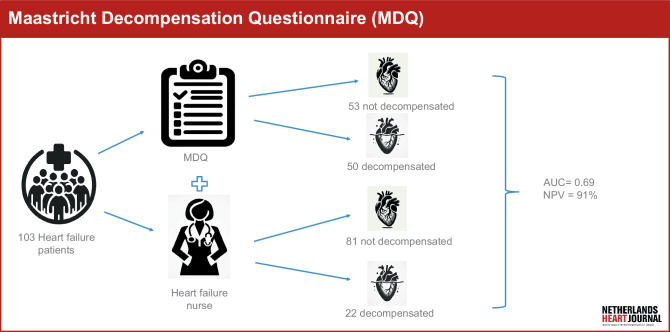
Infographic: Maastricht Decompensation Questionnaire (*AUC* area under the curve, *NPV* negative predictive value)

To address the need for HF readmission prevention and guidance in diuretic therapy, there is a demand for eHealth products that can provide accurate risk assessments of patients’ current fluid status using data that are provided by patients only. In response to this need, we are developing an app that remotely monitors the fluid status of HF patients and provides treatment advice for diuretic therapy in case of deterioration. As a first step in this development, we have created a questionnaire based on expert opinion for evaluating patients’ fluid status using common HF signs and symptoms (Infographic: Fig. 1). The objective of this pilot study is to validate this questionnaire within an ambulant cohort of HF patients, with the ultimate goal of integrating it into an eHealth device for remote monitoring of HF patients.

## Methods

### Development of the Maastricht Decompensation Questionnaire

A working group, comprising three HF cardiologists, two HF nurses and two epidemiologists, collaborated to identify key signs and symptoms for assessing patients’ fluid status. The members defined a set of criteria to evaluate the clinical condition of HF patients, assigned weights to these items based on their severity, and established cut-off values for clinical decompensation. This process was guided by current clinical recommendations and the collective clinical expertise of the participating specialists [20], resulting in the identification of 17 specific items. These items were then translated into a questionnaire known as the Maastricht Decompensation Questionnaire (MDQ), as outlined in Tab. 1. Based on the patients’ response, a score was calculated, allowing for the classification of patients as either ‘decompensated’ (MDQ score ≥ 10) or ‘not decompensated’ (MDQ score < 10). The score per item was chosen based on experience to achieve this threshold to enhance the MDQ’s sensitivity and specificity and prevent potential undertreatment or overtreatment of patients. Additionally, the maximum score for symptoms such as dyspnoea, weight gain and oedema was set at 10, so patients with severe symptoms were directly classified as decompensated. The MDQ questionnaire was made available to all HF patients attending the outpatient HF clinic in Maastricht, the Netherlands, and participation was voluntary. All patients received treatment in accordance with the prevailing European Society of Cardiology HF guidelines [20].

**Table 1:** Maastricht Decompensation Questionnaire

No	Question	Answer
1	I have chest pain	O Yes, at rest (5)
		O Yes, during exercise (3)
		O No (0)
2	The chest pain is worse than usual (answer this question only if your answer to question 1 was ‘Yes, at rest’ or ‘Yes, during exercise’)	O Yes, a lot (5)
		O Yes, a little (3)
		O No (0)
3	I experience shortness of breath	O Yes, at rest (5)
		O Yes, during light exercise (3)
		O Yes, during heavy exercise (1)
		O No (0)
4	The shortness of breath is worse than usual (answer this question only if your answer to question 3 was ‘Yes, at rest’, ‘Yes, during light exercise’ or ‘Yes, during heavy exercise’)	O Yes, a lot (3)
		O Yes, a little (1)
		O No (0)
5	I experience shortness of breath when I tie my shoelaces or pull up my socks	O Yes (2)
		O No (0)
6	My feet, ankles and/or legs are swollen	O Yes, a lot (3)
		O Yes, a little (1)
		O No (0)
7	The swelling of my feet, ankles and/or legs is worse than usual (answer this question only if your answer to question 6 was ‘Yes, a lot’ or ‘Yes, a little’)	O Yes (4)
		O No (0)
8	I feel more bloated or nauseous than usual and/or have less appetite	O Yes (3)
		O No (0)
9	I have gained weight over the past 2 weeks	O Yes, 5 kg (10)
		O Yes, 4 kg (8)
		O Yes, 3 kg (6)
		O Yes, 2 kg (4)
		O Yes 1 kg (2)
		O No (0)
10	I can exercise less intensively than usual	O Yes, very much (3)
		O Yes, a little (1)
		O No (0)
11	I need more time to recover after exercise than usual	O Yes, a lot (3)
		O Yes, a little (1)
		O No (0)
12	I wake up at night because of shortness of breath, dry cough and/or wheezing	O Yes, all 3 symptoms (3)
		O Yes, 2 of the 3 symptoms (2)
		O Yes, 1 symptom (1)
		O No (0)
13	These symptoms are worse than usual (answer this question only if your answer to question 12 was ‘Yes, all 3 symptoms’, ‘Yes, 2 of the 3 symptoms’ or ‘Yes, 1 symptom’)	O Yes, a lot (3)
		O Yes, a little (1)
		O No (0)
14	I experience shortness of breath or dyspnoea when lying down	O Yes (3)
		O No (0)
15	I did not experience shortness of breath or dyspnoea when lying down during the last month (answer this question only if your answer to question 14 was ‘Yes’)	O Yes (3)
		O No (0)
16	I feel more tired than usual	O Yes, a lot (3)
		O Yes, a little (1)
		O No (0)
17	I feel worse than usual	O Yes, a lot (3)
		O Yes, a little (1)
		O No (0)

Number in parentheses represents the score per answer and is not visible to patients

### Data collection

Patients were eligible for inclusion if they were diagnosed with HF across its spectrum and were aged 18 years or older. They were enrolled in the study between May and July 2023. Prior to their scheduled appointments at the outpatient clinic, patients were provided with the MDQ questionnaire and asked to complete it while waiting for their appointments. Subsequently, the assessment of patients’ fluid status, categorised as either decompensated or not decompensated, was carried out by HF nurses who were unaware of the patients’ MDQ scores. Additionally, baseline characteristics of the participants were retrieved from the hospital information system. This investigation received approval from the local ethics committees of Maastricht (METC 2022-3473) and conforms with the principles outlined in the Declaration of Helsinki [21]. Written informed consent was obtained from all subjects during their visit to the outpatient clinic to participate in the study.

### Outcomes

The primary objective of this study was to assess the validity of the MDQ in evaluating fluid status among HF patients, using the HF nurse’s determination as the gold standard. Patients were categorised as ‘classified correctly’ if the fluid status indicated by the MDQ aligned with the judgement of the HF nurse. Conversely, patients were considered ‘classified incorrectly’ in cases where there was a discrepancy between the MDQ assessment and that of the HF nurse. The secondary objective was to determine the MDQ’s reliability by assessing the internal consistency among its items and calculating the corrected item-total correlation (CITC) for each item.

### Sample size calculation

Sample size was calculated using the Cleveland Clinic sample size calculator (riskcalc.org). Based on previous findings from comparable risk models [22], the expected area under the curve (AUC) was set to 0.69. The AUC for the null hypothesis was set to 0.5, the expected prevalence ratio to 0.2 (ratio of positive cases/total sample size). Type I error rate (alpha) was set at 0.05. To yield a power of 0.8, total sample size had to be 98.

### Statistical analysis

To describe baseline characteristics of the participants, descriptive statistics were performed. To analyse differences in baseline characteristics, an independent *t*-test (if data were normally distributed) or Mann-Whitney test (if data were not normally distributed) was used. The performance of the MDQ in distinguishing between decompensated and non-decompensated patients was illustrated through a receiver operating characteristics (ROC) curve and assessed by determining the AUC. Sensitivity and specificity calculations were performed using the predefined cut-off of the MDQ score of 10. In addition, as an exploratory analysis, various scores were tested to identify the optimal cut-off value. The internal validity of the MDQ was assessed using Cronbach’s alpha. Additionally, to evaluate the correlation between individual items and the overall score, the CITC was computed. A *p* < 0.05 was considered statistically significant. All analyses were performed with IBM SPSS 25.0 (IBM, Armonk, NY, USA).

## Results

### Baseline characteristics

We included 103 elderly HF patients (mean age = 74 years, SD = 10 years) with mild to moderate symptoms, 29% of whom were female (Electronic Supplementary Material). In total, 41.7% had HF with reduced ejection fraction; most frequently reported comorbidities were atrial fibrillation (AF, 50.5%) and myocardial infarction (42.7%). All patients were able to read, understand and complete the questionnaire successfully.

Twenty-two patients were identified as decompensated (21%) by the HF nurses. Patients identified as decompensated by HF nurses exhibited a higher New York Heart Association (NYHA) classification, systolic blood pressure and N‑terminal pro-B-type natriuretic peptide (NT-proBNP) levels and were more likely to have AF compared to those classified as not decompensated.

### AUC analysis

In total, the MDQ accurately classified 65 patients (63%) as either decompensated (*n* = 17) or not decompensated (*n* = 48). The questionnaire incorrectly categorised 33 patients (32%) as decompensated, despite HF nurses classifying them as not decompensated. Conversely, 5 patients (5%) were identified as being not decompensated by the MDQ but were considered decompensated by the HF nurse.

**Fig. 2:**
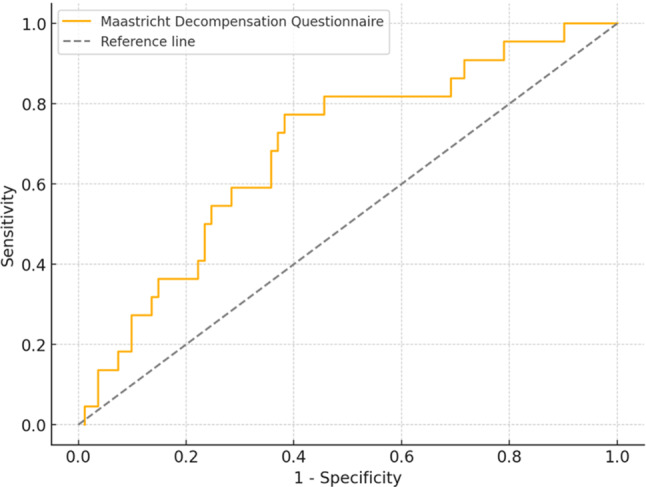
Receiver operating characteristic curve for sensitivity and specificity of the Maastricht Decompensation Questionnaire

Patients who were correctly classified by the questionnaire exhibited a significantly lower NYHA classification (85% NYHA I/II vs 60% NYHA I/II; *p* < 0.05) and were less likely to have hypertension (28% vs 47%; *p* < 0.05) and chronic obstructive pulmonary disease (14% vs 26%; *p* = 0.11) compared to those who were classified incorrectly. The calculated AUC was 0.69 (95% confidence interval (CI): 0.57–0.81; *p* < 0.05). The ROC curve can be seen in Fig. 2.

### Sensitivity and specificity analysis

Using the predefined cut-off value (MDQ ≥ 10), the sensitivity and specificity were determined to be 77.3% and 60.5%, respectively (Tab. 2). With an assumed event prevalence of 21%, this resulted in a positive predictive value of 34.2% and a negative predictive value of 91.0%.

**Table 2:** Diagnostic accuracy of the Maastricht Decompensation Questionnaire (*MDQ*) for different cut-off values

Cut-off value MDQ	Sensitivity (%)	Specificity (%)	PPV (%)	NPV (%)
5	81.8	30.9	24.0	86.5
10	77.3	60.5	34.2	91.0
12	72.7	63.0	34.3	89.7

*NPV* negative predictive value with assumed prevalence of 21%, *PPV* positive predictive value with assumed prevalence of 21%

### Reliability

The calculated Cronbach’s alpha reliability coefficient for the MDQ was found to be 0.85, indicating a strong internal consistency among its items. Among all patients, item no. 9 (‘I gained weight over the past 2 weeks’) exhibited the lowest CITC (0.09), while item no. 11 (‘I need more time to recover after exercise than normal’) had the highest CITC (0.72). For patients classified as decompensated by the HF nurse, item no. 15 (‘I did not experience shortness of breath or dyspnoea when lying down during the last month’) and item no. 9 had the lowest CITC (0.00 and 0.13). Conversely, question no. 4 (‘The shortness of breath is worse than normal’) showed the highest CITC (0.64) in decompensated patients.

## Discussion

Our study suggests that the self-administered MDQ can assist in distinguishing between decompensated and non-decompensated HF patients, which may be a crucial step in identifying those at risk and preventing further deterioration.

### Comparable risk models

Berge et al. assessed the prognostic accuracy of the National Early Warning Score 2, a clinical risk score incorporating various physiological measurements, for predicting mortality in HF patients [23]. They reported an AUC score of 0.65, which was comparable to the prognostic accuracies of high-sensitivity cardiac troponin T (AUC 0.65) and NT-proBNP (AUC 0.67) within their cohort. Kerexeta et al. developed an AI model to predict decompensation in HF patients, utilising signs, symptoms and vital parameters from 488 consecutive HF patients over an average follow-up period of 12.6 months [22]. Their model achieved an AUC score of 0.69, a result that aligns with our findings. In another study, Larburu et al. developed an AI model to identify decompensated HF outpatients, using telemonitoring data including signs, symptoms, vital parameters and clinical data collected from 242 HF patients over a 44-month period [24]. Their model’s performance was also similar to that of ours, with an AUC of 0.67.

Despite the inclusion of larger and more comprehensive datasets in all three trials, along with the use of sophisticated analysis methods, it is noteworthy that these models did not outperform our approach, which relies on a questionnaire based on patient-reported signs and symptoms, in identifying patients at risk. This suggests that the quality of data used in developing the risk model may be just as critical, if not more so, than the quantity of data [25]. HF is a highly heterogeneous syndrome, and the clinical presentation can vary significantly among patients. It is improbable that a single model, often based on retrospective data, can effectively identify all patients at risk. Instead of adhering to a ‘one size fits all’ approach, it is essential to individualise risk monitoring to accommodate the heterogeneity inherent to the HF syndrome. This individualised approach can significantly enhance the diagnostic accuracy of risk models. This principle extends to HF treatment as well, where numerous efforts have been made to customise HF therapy based on patients’ phenotypic profiles. Recognising and addressing the diversity within the HF patient population is crucial for improving both diagnostic accuracy and treatment outcomes [26, 27].

### Individualisation of risk scores

The LINK-HF trial conducted telemedicine-based physiological data collection to predict HF hospitalisation, achieving an AUC of 0.86 for that model [28]. A hundred elderly HF patients with moderate symptoms were enrolled in the study. Initially, patient data, including heart rate, respiratory rate, walking and body posture, among other factors, were collected during a training period to establish individual baselines. Subsequently, alerts were triggered if data deviated from these baselines. This personalised approach yielded promising results.

Despite utilising different input data, our plan includes individualising our model as well. In this context, treating physicians will have the capability to adjust the MDQ according to the patient’s profile. For example, NYHA class III patients often experience dyspnoea during light exercise, leading to higher MDQ scores and potentially more false-positive results. Our findings support this, as patients who were incorrectly classified by the MDQ had significantly higher NYHA classifications compared to those classified correctly. To address these variations, we will conduct an additional trial involving patients with more advanced HF to evaluate the weighting of MDQ questions based on HF severity. Additionally, as the MDQ will be regularly administered, changes in MDQ scores over time will be incorporated into risk determination. These enhancements will be prospectively evaluated in a clinical trial to further refine and validate our approach.

### Additional findings

Evaluating the fluid status of patients with HF poses a considerable challenge due to the limited diagnostic accuracy of most signs and symptoms [29]. The discriminative power of fatigue and weight gain was notably disappointing. Fatigue is a frequently observed symptom that is linked with a range of other medical conditions, and it is not exclusive to HF. Weight gain exhibited a poor CITC, indicating a weak association with total MDQ scores, which is in line with previous findings [30]. This phenomenon could be attributed to the tendency of HF patients, during decompensation, to lose their appetite and develop a catabolic state, resulting in the loss of muscle and fat mass while accumulating fluid weight, thereby maintaining overall weight stability. Consequently, weight may not be suitable for assessing fluid status when monitoring patients. Nevertheless, changes in weight could still prove valuable for monitoring weight loss following the initiation of diuretic therapy.

Ultimately, our study has affirmed that the previously selected MDQ cut-off value (≥ 10) was indeed the most suitable choice in terms of achieving optimal sensitivity and specificity. Additionally, the correctness of our item selection was confirmed by the high internal consistency of the instrument. These outcomes provide strong validation for our expert-based approach and underscore the potential of implementing the MDQ as a valuable tool in clinical practice. By incorporating the MDQ into eHealth interventions, risk prediction can be optimised, enhancing the remote monitoring of HF patients.

### Limitations

In this study, HF nurses assessed patients’ fluid status using standard clinical evaluations, though more precise methods like right heart catheterisation were not used. Still, it is common practice to evaluate fluid status through clinical evaluation without advanced techniques. Independent evaluation of patients’ fluid status might have improved the diagnosis of decompensation. Nevertheless, we found in an earlier trial that there was excellent interobserver reliability in evaluating the clinical status of HF patients among our group of HF nurses. Additionally, patients self-reported weight changes without verification by wearable devices, which could introduce inaccuracies. The MDQ was administered once for clinical evaluation. However, the intention is for the MDQ to be used regularly. Still, most risk scores used in eHealth products were not evaluated before their introduction. Therefore, this pilot validation provides insights for a more evidence-based approach to risk identification in HF patients. Finally, a limited number of psychometric properties of the MDQ have been evaluated, limiting the conclusions we can make about the reliability and validity of the questionnaire. Future trials are necessary to clarify this.

## Conclusion

The MDQ can facilitate the identification of decompensated HF patients through clinical signs and symptoms. Further validation with a larger sample size is warranted to establish the questionnaire’s robustness and generalisability. Customising the MDQ to match the patient’s phenotypic profile may potentially enhance its discriminatory capability, a matter that warrants evaluation in future prospective clinical trials.

## Supplementary Information

Baseline characteristics
