# Supplementary material for: Fluid status assessment in heart failure patients: pilot validation of the Maastricht Decompensation Questionnaire
Source: Neth Heart J. 2024 Dec 10;33(1):7–13. doi: 10.1007/s12471-024-01921-4 (PMC11695504; doi:10.1007/s12471-024-01921-4)
Supplement: Supplementary file 1 — Baseline characteristics [file 12471_2024_1921_MOESM1_ESM.docx]

**Baseline characteristics**

| **Variable** | **n** | **Overall** | **n** | **Decompensated** (HF nurse decision) | **n** | **Not decompensated**  (HF nurse decision) |
| --- | --- | --- | --- | --- | --- | --- |
| Age | 103 | 74.0 ± 10.1 | 22 | 74.9 ± 14.0 | 81 | 73.8 ± 8.9 |
| Gender | 103 |  | 22 |  | 81 |  |
| Male |  | 73 (70.9) |  | 16 (72.7) |  | 57 (70.4) |
| Female |  | 30 (29.1) |  | 6 (27.3) |  | 24 (29.6) |
| NYHA classification | 76 |  | 13 |  | 63 |  |
| NYHA I |  | 14 (18.4) |  | 0 (0.0) |  | 14 (22.2) |
| NYHA II |  | 44 (57.9) |  | 8 (61.5) |  | 36 (57.1) |
| NYHA III |  | 18 (23.7) |  | 5 (38.5) |  | 13 (20.6) |
| NYHA IV |  | 0 (0.0) |  | 0 (0.0) |  |  |
| Heart rhythm at baseline | 103 |  | 22 |  | 81 |  |
| Sinus rhythm |  | 59 (57.3) |  | 10 (45.5) |  | 49 (60.5) |
| Atrial fibrillation |  | 25 (24.3) |  | 9 (40.9) |  | 16 (19.8) |
| Pacemaker rhythm |  | 19 (18.4) |  | 3 (13.6) |  | 16 (19.8) |
| Heart rate | 102 | 70 (62-81) | 22 | 71 (65-84) | 80 | 70 (61-79) |
| LV ejection fraction | 103 | 41.7 ± 11.1 | 22 | 43.1 ± 11.5 | 81 | 41.3 ± 11.0 |
| Type of HF | 103 |  | 22 |  | 81 |  |
| HFrEF |  | 43 (41.7) |  | 8 (36.4) |  | 35 (43.2) |
| HFmrEF |  | 37 (35.9) |  | 9 (40.9) |  | 28 (34.6) |
| HFpEF |  | 23 (22.3) |  | 5 (22.7) |  | 18 (22.2) |
| BMI | 99 | 27.4 ± 5.0 | 22 | 27.6 ± 5.0 | 77 | 27.4 ± 5.0 |
| Systolic blood pressure | 97 | 128 (113-144) | 21 | 143 (118-157) | 76 | 121 (112-140) |
| Laboratory data |  |  |  |  |  |  |
| NT-proBNP (pmol/l) | 85 | 95 (33-272) | 18 | 222 (52-333) | 67 | 87 (26-229) |
| Potassium (mmol/l) | 102 | 4.6 ± 0.5 | 21 | 4.5 ± 0.6 | 81 | 4.7 ± (0.5) |
| Sodium (mmol/l) | 102 | 140.7 ± 2.9 | 21 | 141.2 ± 3.2 | 81 | 140.6 ± 2.9 |
| Creatinine (umol/l) | 102 | 124.3 ± 43.0 | 21 | 137.6 ± 39.1 | 81 | 120.9 ± 43.5 |
| Hemoglobin (mmol/l) | 94 | 8.6 ± 1.1 | 19 | 8.7 ± 1.2 | 75 | 8.5 ± 1.1 |
| Medication |  |  |  |  |  |  |
| Beta-blocker | 103 | 91 (88.3) | 22 | 19 (86.4) | 81 | 72 (88.9) |
| ACE-inhibitor | 103 | 26 (25.2) | 22 | 5 (22.7) | 81 | 21 (25.9) |
| AT2 antagonist | 103 | 20 (19.4) | 22 | 5 (22.7) | 81 | 15 (18.5) |
| ARNI | 103 | 40 (38.8) | 22 | 7 (31.8) | 81 | 33 (40.7) |
| Diuretics | 103 | 72 (69.9) | 22 | 17 (77.3) | 81 | 55 (67.9) |
| SGLT-2 inhibitor | 103 | 60 (58.3) | 22 | 10 (45.5) | 81 | 50 (61.7) |
| MRA | 103 | 70 (68.0) | 22 | 13 (59.1) | 81 | 57 (70.4) |
| Lanoxin | 103 | 11 (10.7) | 22 | 4 (18.2) | 81 | 7 (8.6) |
| Device | 103 |  | 22 |  | 81 |  |
| Pacemaker |  | 21 (20.4) |  | 3 (13.6) |  | 18 (22.2) |
| ICD |  | 13 (12.6) |  | 0 (0) |  | 13 (16.0) |
| Comorbidities | 103 |  | 22 |  | 81 |  |
| Hypertension |  | 36 (35.0) |  | 11 (50) |  | 25 (30.9) |
| COPD |  | 19 (18.4) |  | 5 (22.7) |  | 14 (17.3) |
| Diabetes mellitus |  | 26 (25.2) |  | 5 (22.7) |  | 21 (25.9) |
| Aortic valve stenosis |  | 9 (8.7) |  | 3 (13.6) |  | 6 (7.4) |
| Myocardial infarction |  | 44 (42.7) |  | 10 (45.5) |  | 34 (42.0) |
| CABG |  | 13 (12.6) |  | 4 (18.2) |  | 9 (11.1) |
| CVA/TIA |  | 15 (14.6) |  | 4 (18.2) |  | 11 (13.6) |
| Atrial fibrillation |  | 52 (50.5) |  | 14 (63.6) |  | 38 (46.9) |

Values are presented as number (%) or mean ± SD; ┼ = median (interquartile range (IQR) 25–75). ACE = angiotensin-converting-enzyme; ATII = Angiotensin II; ARNI = angiotensin receptor/neprilysin inhibitor; BMI = Body mass index; CABG = coronary artery bypass grafting; COPD = chronic obstructive pulmonary disease; CVA = cerebrovascular accident; n = number; HFmrEF = heart failure with mildly reduced ejection fraction; HFpEF = heart failure with preserved ejection fraction; HFrEF = heart failure with reduced ejection fraction; ICD = implantable cardioverter-defibrillator; LV = left-ventricular; NYHA = New York Heart Association Functional Classification; NT-proBNP = N-terminal pro-brain natriuretic peptide; SGLT-2 = Sodium-glucose cotransporter-2; TIA = Trans Ischemic Attack.
